# UVA Induced Oxidative Stress Was Inhibited by Paeoniflorin/Nrf2 Signaling or PLIN2

**DOI:** 10.3389/fphar.2020.00736

**Published:** 2020-05-15

**Authors:** Yan-Song Lu, Yuan Jiang, Jin-ping Yuan, Shi-Bin Jiang, Yang Yang, Pei-yao Zhu, Yu-zhe Sun, Rui-qun Qi, Tao Liu, He-Xiao Wang, Yan Wu, Xing-Hua Gao, Hong-duo Chen

**Affiliations:** ^1^Key Laboratory of Immunodermatology, Ministry of Education, Department of Dermatology, The First Hospital of China Medical University, Shenyang, China; ^2^Department of Internal Medicine, School of Nursing, Shandong University of Traditional Chinese Medicine, Jinan, China; ^3^Department of Thoracic Surgery, the First Hospital of China Medical University, Shenyang, China; ^4^Department of Dermatology, Dermatology Hospital of Southern Medical University, Guangzhou, China; ^5^Department of Urinary Surgery, the First Hospital of China Medical University, Shenyang, China

**Keywords:** UVA, oxidative stress, paeoniflorin, Nrf2, PLIN2

## Abstract

Photodamages caused by UVA radiation induced oxidative injuries are closely related to photoaging and skin cancer. Paeoniflorin (PF), extracted from the root of Paeonia lactiflora, has been reported to be an effective antioxidant. PLIN2, known as adipose differentiation-related protein, has been previously involved in the regulation of oxidative stress. In this study, we were sought to investigate the photo-protective property of PF and PLIN2 in UVA-radiated human dermal fibroblasts (HDFs). HDFs were pre-treated with PF (800 μM) followed by UVA radiation (22.5 J/cm2). MTS activity, cell apoptosis, ROS, MDA, and SOD were detected, respectively. The expressions of Nrf2, HO-1, NQ-O1, and PLIN2 were determined using RT-qPCR or western blot. Nrf2 was silenced by siRNA, and PLIN2 was overexpressed *via* lentiviral transduction. Comparing to the UVA radiation, PF pre-treatment could prominently increase the MTS activity, decrease cell apoptosis, reduce the generations of ROS and MDA, increase the activity of SOD and increase the expression of Nrf2 and its target genes HO-1 and NQ-O1. When Nrf2 was knocked down, PF lost above protective properties. In addition, UVA induced oxidative stress led to upregulation of PLIN2 and the latter could be decreased by PF. Overexpression of PLIN2 improved MTS activity and reduced MDA level in HDFs. The combination of PLIN2 overexpression and PF pre-treatment corporately inhibited UVA-induced injury. Besides, we also found that PF and PLIN2 had a compensatory protection against UVA induced oxidative stress. In conclusion, our study demonstrated that UVA induced photodamages could be inhibited by PF *via* Nrf2/HO-1/NQ-O1 signaling pathway or by PLIN2, and the combination of PLIN2 overexpression and PF played additive effects against UVA-related oxidative stress.

## Introduction

Ultraviolet A (UVA) radiation (315–400 nm) is a major environmental challenge to the skin (Pinnell, 2003; Bachelor and Bowden, 2004). As the consequence of UVA over-exposure, skin cells produce reactive oxygen species (ROS) including singlet oxygen, superoxide anions, and hydrogen peroxide. ROS interact with lipid-rich membranes, enzymes, and cellular DNA and may change their structures and interfere with their functions. Accumulated oxidative injuries in skin cells are major causes of photodamages, photoaging, and photo-carcinogenesis (D’Orazio et al., 2013; Gilchrest, 2013; Kammeyer and Luiten, 2015). Some studies have also revealed that UVA generated ROS may cause lipid peroxidation, apoptosis, and upregulation of nuclear factor erythroid 2-like 2 (Nrf2), an oxidative stress marker (Kulms et al., 2002; Hirota et al., 2005; Hseu et al., 2012). The equipment of complex anti-oxidant mechanisms enables skin cells to inhibit oxidative stress. Nrf2 transcription factor was critical in protecting the skin cells against oxidative stress and UVA radiation (Hirota et al., 2005), and the antioxidative ability of Nrf2 has been confirmed in keratinocytes, melanocytes, and skin fibroblasts (Marrot et al., 2008; Raval et al., 2012; Schafer and Werner, 2015). Nrf2 could be activated by various factors, such as various external injury stimuli and plant-derived polyphenols protective stimuli (Kim et al., 2010; Baird and Dinkova-Kostova, 2011). Upon activation, Nrf2 dissociates from keap1 and translocates into the nucleus, where it binds to antioxidant response element (ARE) and activates the promoter region of many antioxidative genes, such as heme oxygenase 1 (HO-1) and NAD(P)H quinone oxidoreductase 1 (NQO1) (Sasaki et al., 2000; Meewes et al., 2001; Surh, 2003; Kim et al., 2010).

Accumulating studies have focused on the antioxidative functions of certain natural compounds, such as zerumbone and Stachys riederi (Saewan and Jimtaisong, 2015; Yang et al., 2018; Hwang et al., 2019). Paeoniflorin (PF), likewise, has also been reported to be an effective anti-oxidant (Shou et al., 2018; Liu et al., 2019; Wang et al., 2019). PF is a main bioactive component of a traditional Chinese medicine, the total glucosides of paeony, and is a monoterpene glucoside compound extracted from the root of Paeonia lactiflora (Chen et al., 2004). Clinical data showed its wide utility in treating autoimmune diseases, such as rheumatoid arthritis and psoriasis (Zhang and Dai, 2012; Yu et al., 2017). PF has been reported to be an essential antioxidant that protected human retinal pigment epithelium from H₂O₂-induced oxidative injury (Wankun et al., 2011). Pre-treatment of PF protected neural cells by decreasing the production of ROS and malondialdehyde (MDA), and increasing the activity of superoxide dismutase (SOD) (Mao et al., 2010). PF has also been proved to protect human pulmonary endothelial cells from radiation-induced oxidative injury through the Nrf2/HO-1 pathway (Yu et al., 2013). Furthermore, PF could activate the Nrf2/ARE pathway to protect Schwann cells from oxidative stress injury (Yang et al., 2016). Although there was evidence that PF could protect keratinocytes from Ultraviolet B (UVB) induced cell damages, no studies have revealed the interactions between PF, UVA, and skin cells (Kong et al., 2016).

Lipid-binding protein perilipin 2 (PLIN2), also known as adipose differentiation-related protein (ADRP), regulates the formation of lipid droplets (LDs), uptake of fatty acid and storage of lipid, and is expressed in multiple nonadipose tissues (Magne et al., 2013; Graffmann et al., 2016; Son et al., 2016). PLIN2 has been suggested to be associated with oxidative stress. In hepatocytes, PLIN2 could be upregulated by chemical induced ROS (Jin et al., 2018; Chen et al., 2019). Chemical associated oxidative stress has been reported to induce increase in PLIN2 which in turn protected cells against ROS and preserved the mitochondrial integrity in breast cancer cells (Cadenas et al., 2019). However, little is known about the impact of UVA-related oxidative stress on the expression and function of PLIN2 in human dermal fibroblasts (HDFs).

In this study, we investigated the anti-photodamage potential and the underlying mechanism of PF in HDFs. The expression levels of PLIN2 induced by UVA alone or in combination with PF pre-treatment and the functions of PLIN2 were also detected. We may provide potential antiphotodamage therapeutic strategy.

## Materials and Methods

### Cell Culture

The studies involving human participants were reviewed and approved by Ethics Committee of Medical Science Research, the First Hospital of China Medical University. Ethics number: AF-SOP-07-1.0-01. Written informed consent to participate in this study was not required in accordance with local/national guidelines. Primary HDFs were isolated from circumcised foreskins of six young individuals aged between 20 and 25 years old. Cells were grown in Dulbecco’s Modified Eagle Medium (DMEM, Biological Industries, Israel). Supplementary fetal bovine serum (FBS, Biological Industries, Israel) containing 1% penicillin and streptomycin (Biological Industries, Israel) were also added into the culture medium. Cells were maintained in an incubator with the atmosphere of 37°C and 5% CO_2_.

### UVA Radiation

Cells were washed by phosphate-buffered saline (PBS) before being exposed to UVA radiation (22.5 J/cm^2^) using the UV 801 KL equipment (Waldmann, Germany). During radiation, cells were in a thin layer of cool PBS and the distance between the cells and the lamp was 15cm. After radiation, PBS was replaced by fresh medium, and cells were re-cultured for the subsequent experiments. All experiments were tested at least three times.

### MTS Assay

The effects of PF and UVA radiation on cell injury were assessed using the CellTiter 96® Aqueous Non-Radioactive Cell Proliferation Assay (MTS assay, Promega, USA) (Chignell and Sik, 2003; Roberts et al., 2008; Zhao et al., 2009; Yin et al., 2012; Dolat et al., 2020). After cell seeding, the fibroblasts (2000 cells/well, in a 96-well plate) were pre-treated with PF (Sigma-Aldrich, USA, purity ≥ 98%) for 24 h before UVA radiation. MTS solution was added into cells at different time points, and the MTS activity was calculated by measuring the absorbance at the wavelength of 490 nm by a microplate reader 3 h later.

### Flow Cytometry Assay

Cell apoptosis was measured by flow cytometry using a BD LSRFortessa instrument (BD Bioscience, USA) and Annexin V-FITC Apoptosis Detection Kit (BD Biosciences, USA). The cells were divided into three groups, including control group, UVA group, and PF pre-treated group. Cells were trypsinized, and washed with cold PBS. After centrifugation, cells were incubated with 5-μl propidium iodide (PI) and 5-μl annexin V in dark following manufacturer’s instructions. The results were presented as the percentage of apoptotic cells.

### Detection of ROS Generation

The generation of intracellular ROS was detected by fluorescence microscopy (Leica, Germany) using Reactive Oxygen Species Assay Kit (Beyotime, China). After PF pre-treatment and UVA radiation, fibroblasts were incubated with 10 μM dicholofluorescein (DCFH2-DA) in DMEM without FBS at 37°C for 20 min. Intracellular ROS could transform DCFH-DA into fluorescent compound 2,7-dichlorofluorescein (DCF) and emit green fluorescent. The production of ROS was indicated by DCF fluorescence under fluorescence microscope. The fluorescence intensity was quantified using Image-Pro Plus 6.0 in three randomly visual fields.

### Measurement of MDA and SOD

Cells were homogenized in RIPA lysis buffer (Beyotime, China) on ice, and lysed cells were centrifuged at 15,000 *g* for 15 min at 4°C. The supernatant was then collected and subjected to the measurement of protein contents, MDA levels and SOD activity, according to the manufacturer’s instructions. The protein concentration was detected by BCA Protein Assay Kit (Beyotime, China). The level of MDA was measured using Lipid Peroxidation MDA assay kit (Beyotime, China), and the results were shown as nmol/mg protein. The activity of SOD was detected by Total Superoxide Dismutase Assay Kit with WST-8 (Beyotime, China), and the results were presented as U/mg protein.

### Real-Time Quantitative PCR (RT-qPCR) Assay

Total RNA was extracted using the miRNeasy Mini Kit (Qiagen, Germany) following the manufacturer’s procedure. Total RNA (1 μg) was used to synthesize complementary DNA (cDNA) using a GoScript Reverse Transcription Kit (Promega, USA) at 42°C for 15 min and 70°C for 15 min as the protocols supplied. Primer sequences for Nrf2, PLIN2, and GAPDH were designed using Primer-Primier 6.0 software. The primer sequences for PCR reaction were shown as follows:

Nrf2-f, 5′-CTTGGCCTCAGTGATTCTGAAGTG-3′;
Nrf2-r, 3′-CCTGAGATGGTGACAAGGGTTGTA-5′;
PLIN2-f, 5′-CACAACCGAGTGTGGTGACT-3′;
PLIN2-r, 3′- CACACCGTTCTCTGCCATCT-5′;
GAPDH-f, 5′-TGGAGTCTACTGGCGTCTT-3′;
GAPDH-r, 5′-TGTCATATTTCTCGTGGTTCA-3′.

Each reaction mixture (20 ul) contained each primer (0.4 ul), 2× qPCR Master Mix (10 ul) (RT2 SYBR Green qPCR Mastermix, Promega, USA), cDNA (2 ul), and nuclease-free water. The expression of mRNA was quantified with the 7900HT Fast Real-Time PCR System (Applied Biosystems, USA). Reaction condition was performed as follows: 95°C for 2 min; 40 cycles of 95°C for 15 s; 60°C for 1 min. Melting curves were generated to confirm synthesis specificity. The 2^−ΔΔCt^ method was employed to calculate the relative expression levels of target genes, and GAPDH was used as the control gene. All experiments were performed at least three times.

### Western Blot Analysis

Cells were harvested and lysed. BCA protein assay kit was used to measure the concentration of proteins. Equal amount of denatured proteins (35 μg) were loaded into corresponding lanes, separated by 12% SDS-polyacrylamide gel electrophoresis (Beyotime, China) and then electro-transferred onto polyvinylidene difluoride (PVDF) membranes (Merck Millipore, Germany). The membranes were blocked with 5% non-fat dried milk in TBST containing 1% Tween-20 for 2 h at room temperature, and then incubated with the primary antibodies overnight at 4°C. On the second day, the membranes were incubated with secondary antibodies, then washed. After the incubation with enhanced chemiluminescence reagents (Beyotime, China), the blots were detected by using MicroChemi™ Chemiluminescent Imaging System (DNR Bio-Imaging Systems, Israel). The result was quantified by using Image-Pro Plus 6.0. All values were normalized to the internal control of GAPDH. All experiments were performed at least three times.

Primary antibodies included anti-GAPDH (60004-1-1g, Proteintech, China), anti-Nrf2 (ab137550, Abcam, UK), anti-HO-1 (5853S, Cell Signaling Technology, USA) anti-NQ-O1 (ab28947, Abcam, UK), and anti-PLIN2 (ab52356, Abcam, UK) antibodies. The secondary antibody was a HRP-conjugated goat anti-rabbit or an anti-mouse IgG polyclonal antibody (Beyotime, China).

### SiRNA Transfection

Fibroblasts were grown to 40–60% confluence in a 6-well plate in culture medium without antibiotics before transfection. The control siRNA or Nrf2 siRNAs (ORIGENE, USA) were transfected at a final concentration of 10 nM. Lipofectamine RNAiMAX (Invitrogen, USA) was used as the transfection reagent. For each transfection, siRNA and RNAiMAX were diluted with Opti-MEM (Gibco, USA) before mixing. The siRNA/RNAiMAX mixture (200 μl) was incubated at room temperature for 5 min to allow complex formation. Subsequently, the siRNA/RNAiMAX mixture was added to the cells in the 6-well plates with 1.8-ml Opti-MEM. The transfection medium was replaced with DMEM after incubation for 6 h. The transfection efficiency was detected by RT-qPCR and western blot at 24, 48, and 72 h after transfection. Two specific siRNA sequence were shown as follows:

Nrf2 siRNA1 - rArUrUrGrArUrGrUrUrUrCrUrGrArUrCrUrArUrCrArCrUTT
Nrf2 siRNA2 - rGrUrCrArGrUrArUrGrUrUrGrArArUrCrArGrUrArGrUrUTC.

### Overexpression of PLIN2 by Lentiviral Transduction

Primary fibroblasts were cultured in antibiotics-free medium before lentiviral transfection. The cells were transfected with an empty vector (Lenti-EGFP) or lentivirus containing EGFP-PLIN2 (Lenti-PLIN2) in transfection reagent Polybrene. All lentiviruses and the polybrene were manufactured by OBiO Technology (shanghai) Corp., Ltd. (China). Puromycin (Solarbio, China; 1.0 μg/ml) was used to screen fibroblasts which were successfully transfected. Successful transfection was confirmed by observing EGFP positive cells under an inverted fluorescent microscope and the transduction efficiency was detected by RT-qPCR.

### Detection of DNA Synthesis

DNA synthesis was detected by Cell-Light Apollo488 EdU Stain Kit (Ribobio, China) following the manufacturer’s instructions. In brief, cells were incubated with 50 μmol/L 5-Ethynyl-221 2’- deoxyuridine (EdU) dissolved in DMEM for 2 h, and then washed with PBS for three times. Cells were fixed with 4% paraformaldehyde and permeated with 0.5% Triton-X100. The Apollo Stain Kit and DAPI (Solarbio, China) were used to stain EdU and nuclei, respectively. The proportion of EdU positive (EdU^+^) cells was shown as the number of EdU^+^ cells against total number of cells counted in three randomly visual fields under a fluorescence microscope.

### Statistical Analysis

Statistical analysis was performed using SPSS v22.0 (USA). One-way analysis of variance (ANOVA) was used for multiple group comparisons, and Bonferroni multiple comparisons test was used for the comparison of two groups. The results were shown as mean ± standard deviation (mean ± SD) from at least three independent experiments. A *p-*values < 0.05 was considered to be statistically significant.

## Result

### PF Inhibited UVA-Induced Injuries in HDFs

**Figure 1:**
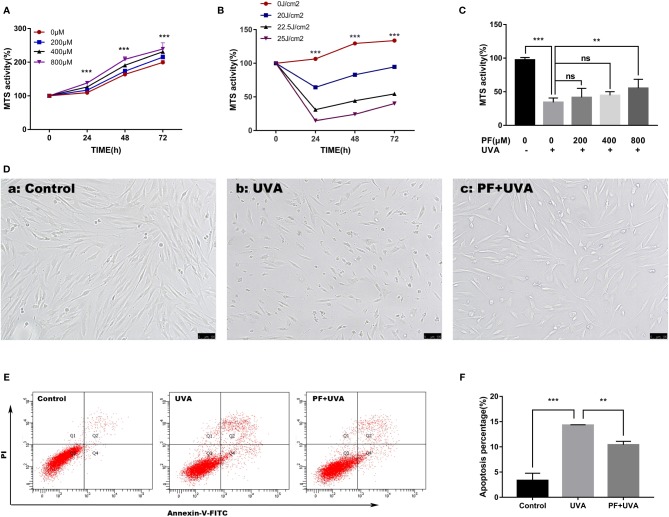
PF suppressed UVA-induced injuries in HDFs. **(A)** MTS assay on the cytotoxicity of PF (0, 200, 400, and 800 μM) in fibroblasts at 24, 48, and 72 h. **(B)** MTS assay on the cytotoxicity of UVA (0, 20, 22.5, and 25 J/cm^2^) in fibroblasts at 24, 48, and 72 h. **(C)** MTS assay detected the cytoprotective function of PF (0, 200, 400, and 800 μM) against UVA (22.5 J/cm^2^) in fibroblasts. **(D)** Cellular morphology of fibroblasts with different treatments visualized by inverted microscope. **(E)** The cells apoptosis was detected by using flow cytometry with annexin-V-FITC and PI staining. The Q3 quadrant contained the vital population; the Q1 quadrant contained the death cells; the Q2 quadrant contained the late apoptotic cells; the Q4 quadrant contained the early apoptotic cells. **(F)** The data represented the apoptosis percentage containing the Q2 and Q4 quadrants in each group. All data were expressed as the mean ± SD according to at least three independent experiments. ^**^p < 0.01, ^***^p < 0.001, ^ns^*p >* 0.05.

The cytotoxicity of PF on HDFs was examined by MTS. Cells were treated with different concentrations of PF (0, 200, 400, 800 μM) and MTS activity was detected at 24, 48, and 72 h. As shown in **Figure 1A**, we found that PF had no cytotoxicity to the fibroblasts. On the other hand, fibroblasts were exposed to different doses of UVA (0, 20, 22.5, 25 J/cm^2^), and we found that UVA radiation could induce cell injury in a dose-dependent manner (**Figure 1B**). UVA at doses of 22.5 and 25 J/cm^2^ both effectively reduced the MTS activity, and UVA at the dose of 22.5 J/cm^2^ was ultimately explored in our study. The cytoprotective function of PF was then evaluated. In results, pre-treatment of PF at a concentration of 800 μM could significantly neutralize the cytotoxicity of UVA (22.5 J/cm^2^), but PF at concentrations of 200 and 400 μM had no significant impact (**Figure 1C**).

The cytoprotective properties of PF against UVA in HDFs were initially observed under the inverted microscope. As shown in **Figure 1D(b)**, the cell number was significantly decreased and cells became deformed and shrunk after UVA radiation comparing to the control group (**Figure 1D(a)**), and these changes could be reversed by PF pre-treatment (**Figure 1D(c)**). Similarly, in the detection of apoptosis by flow cytometry (**Figures 1E, F**), we observed that the percentage of apoptotic cells was significantly increased to 14.3% under the UVA radiation only, which was much less (10.4%) in cells treated with both PF and UVA. Taken together, our results suggested PF pre-treatment could prominently alleviate the cytotoxicity of UVA radiation in fibroblasts.

### PF Prevented UVA-Induced Oxidative Stress in HDFs

**Figure 2:**
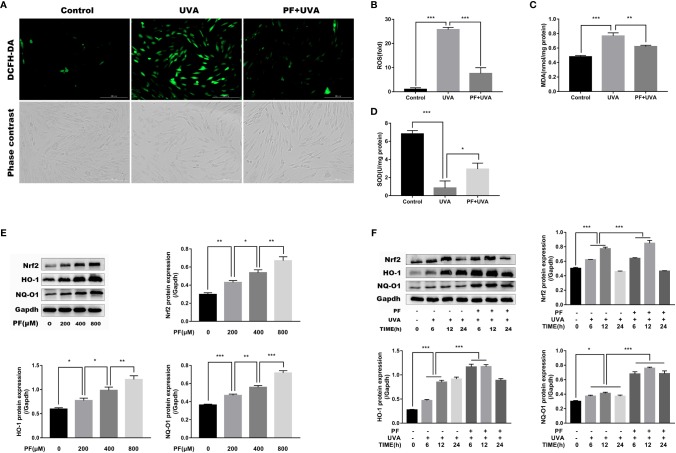
PF inhibited UVA-induced oxidative stress in HDFs. **(A)** The levels of ROS were indicated by DCF fluorescence emitting green fluorescent under fluorescence microscope. **(B)** The DCF fluorescence intensity was quantified by Image-Pro Plus 6.0. and the intensity of DCF fluorescence in each group was normalized to the control group. Detection of the generation of MDA **(C)** and the activity of SOD **(D)** in each group. **(E)** Western blot showing the protein levels of Nrf2, HO-1, and NQ-O1 in fibroblasts treated with PF (0, 200, 400, and 800 μM), the representative result of western blot and the quantitative analyses of three independent experiments were shown. **(F)** The effects of UVA and PF pre-treatment +UVA on the protein expressions of Nrf2, HO-1, and NQ-O1 at time points of 6, 12, and 24 h, the representative result of western blot and the quantitative analyses of three independent experiments. All data were expressed as the mean ± SD according to at least three independent experiments. ^*^p < 0.05, ^**^p < 0.01, ^***^p < 0.001.

Excessive UVA radiation could lead to ROS generation and cell death (Hseu et al., 2015; Yang et al., 2018). Therefore, we detected whether PF could attenuate ROS formation induced by UVA in fibroblasts. As shown in **Figures 2A, B**, cells treated with UVA alone displayed dramatically increase of DCF fluorescence compared with the control group, whereas additional application of PF appeared to diminish increased DCF fluorescence induced by UVA, suggesting PF pre-treatment may protected fibroblasts from UVA induced ROS production.

The representative markers of oxidative stress, MDA and SOD were then detected. As shown in **Figures 2C, D**, UVA has significantly increased the level of MDA and decreased the activity of SOD, and pre-treatment of PF has reversed the impact of UVA on MDA and SOD. Taken together, PF might inhibit the oxidative stress injury induced by UVA radiation in fibroblasts.

### PF Activated Nrf2 and Upregulated Antioxidant Genes in HDFs

Having shown that PF could protect fibroblasts from UVA induced oxidative stress, we speculated if Nrf2/HO-1/NQ-O1 signaling pathway was involved in this process. Western blot assay revealed that the protein levels of Nrf2, HO-1, and NQ-O1 were increased in PF treated cells in a concentration dependent manner (**Figure 2E**). In aspect of UVA stimulation, these three proteins were all gradually upregulated up to 12 h after UVA radiation, and downregulated again after 24 h. In addition, pre-treatment of PF and UVA were likely to corporately increase the expressions of these three proteins 12 h after the UVA radiation (**Figure 2F**). These results have provided evidence of the participation of the Nrf2/HO-1/NQ-O1 signaling in the protection of HDFs against UVA induced oxidative stress by PF.

### Nrf2 Knockdown Obliterated the Protective Property of PF Against UVA-Induced Oxidative Stress and Cytotoxicity

**Figure 3:**
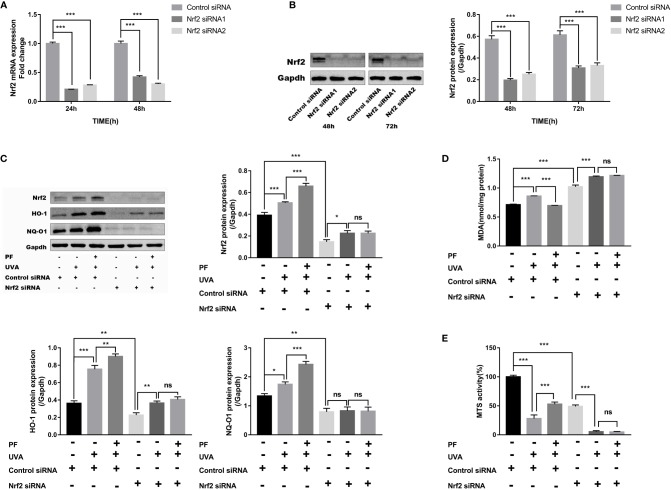
PF inhibited UVA-induced oxidative stress and cytotoxicity *via* Nrf2. **(A)** The mRNA levels of Nrf2 in fibroblasts at 24 and 48 h of transfection. The relative expression level of Nrf2 in each group was normalized to the control group. **(B)** The protein levels of Nrf2 in fibroblasts at 48 and 72 h of transfection, the representative result of western blot and the quantitative analyses of three independent experiments were shown. **(C)** Western blot detection of the protein levels of Nrf2, HO-1, and NQ-O1 after UVA and PF-pretreatment in absent of Nrf2, the representative result of western blot and the quantitative analyses of three independent experiments were shown. **(D)** Detection of MDA level in fibroblasts treated with UVA radiation and PF in absence of Nrf2. **(E)** The MTS activity of fibroblasts with UVA radiation and PF-pretreatment in absence of Nrf2. All data were expressed as the mean ± standard deviation according to at least three independent experiments. ^*^p < 0.05, ^**^p < 0.01, ^***^p < 0.001, ^ns^*p >* 0.05.

To detect whether the antioxidant property of PF was mediated by Nrf2, fibroblasts were transfected with Nrf2 siRNA. Successful knockdown of Nrf2 was confirmed by RT-qPCR and western blot analysis (**Figures 3A, B**). Although two siRNAs successfully knocked down Nrf2 at protein level, siRNA1 failed to consistently inhibit the Nrf2 mRNA expression, siRNA2 was therefore selected for the following experiments. As expected, Nrf2 knockdown abrogated the increase of NQ-O1 and HO-1 in PF pre-treatment +UVA comparing to UVA radiation (**Figure 3C**).

We have also detected increased production of MDA and decreased MTS activity when Nrf2 was knocked down. Although UVA appeared to further increase the MDA level and decrease the MTS activity in Nrf2 silenced cells, PF was unable to alter these impacts of UVA (**Figures 3D, E**). These data suggested that PF may exerted its antioxidant function *via* Nrf2.

### PLIN2 Was Regulated by UVA and PF

**Figure 4:**
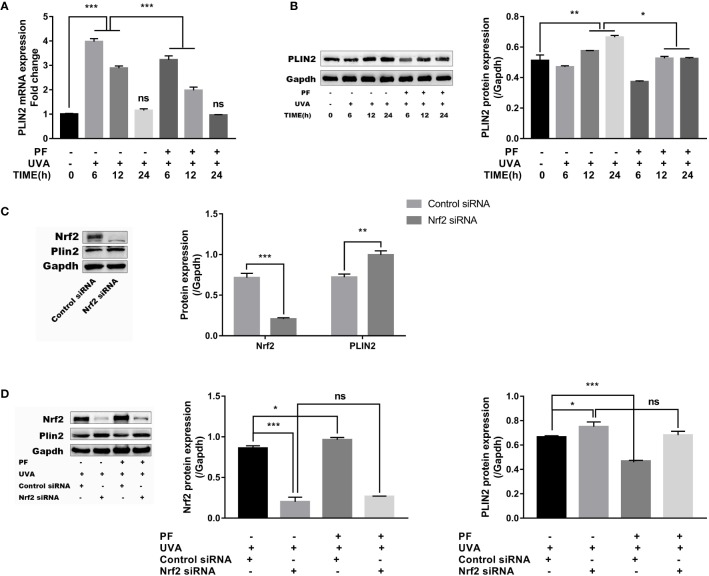
Regulation of PLIN2 by UVA and PF. **(A)** The effects of UVA and PF pre-treatment +UVA on the mRNA expression of PLIN2 tested by RT-qPCR at time points of 6, 12, and 24 h. **(B)** The effects of UVA and PF pre-treatment +UVA on the protein expressions of PLIN2 tested by Western blot at time points of 6, 12, and 24 h. **(C)** The protein expression of PLIN2 after Nrf2 was knocked down in fibroblasts. **(D)** The protein expression of PLIN2 in fibroblasts with UVA radiation and PF-pretreatment in absence of Nrf2. The representative result of western blot and the quantitative analyses of three independent experiments were shown **(B, D)**. All data were expressed as the mean ± SD according to at least three independent experiments. ^*^p < 0.05, ^**^p < 0.01, ^***^p < 0.001, ^ns^*p >* 0.05.

PLIN2 has been previously proved to be involved in the regulation of oxidative stress, therefore, we speculated PLIN2 may be associated with the regulation of oxidative stress stimulated by UVA radiation. We initially detected the expression level of PLIN2 under UVA radiation or PF pre-treatment + UVA. As shown in **Figure 4A**, mRNA level of PLIN2 was significantly upregulated soon after 6 h of UVA radiation, and gradually decreased to the normal level after 24 h. UVA increased PLIN2 expression was downregulated by PF pre-treatment at 6 and 12 h after UVA radiation. When we detected the protein level of PLIN2, we found that PLIN2 was increased after 12 and 24 h of UVA exposure, and at each time point PF pre-treated cells showed less PLIN2 expression (**Figure 4B**). Our findings have shown that UVA alone or in combination with PF pre-treatment could regulate PLIN2 expression, implying PLIN2 may be associated with UVA and PF by regulating oxidative stress.

Nrf2 was then knocked down to further investigate the relationships between PLIN2 and oxidative stress in fibroblasts. In results, we have detected that Nrf2 silencing could lead to increase of PLIN2 expression (**Figure 4C**). Upregulation of UVA induced PLIN2 expression was also strengthened by Nrf2 silencing. It was interesting to see that PF pre-treatment failed to alter the impact of UVA on the PLIN2 expression in Nrf2 silenced cells, possibly because PF was unable to protect fibroblasts against UVA induced oxidative stress in the absence of Nrf2 (**Figure 4D**).

### PLIN2 Overexpression Promoted Cell Proliferation and Inhibited Oxidative Stress in HDFs

**Figure 5:**
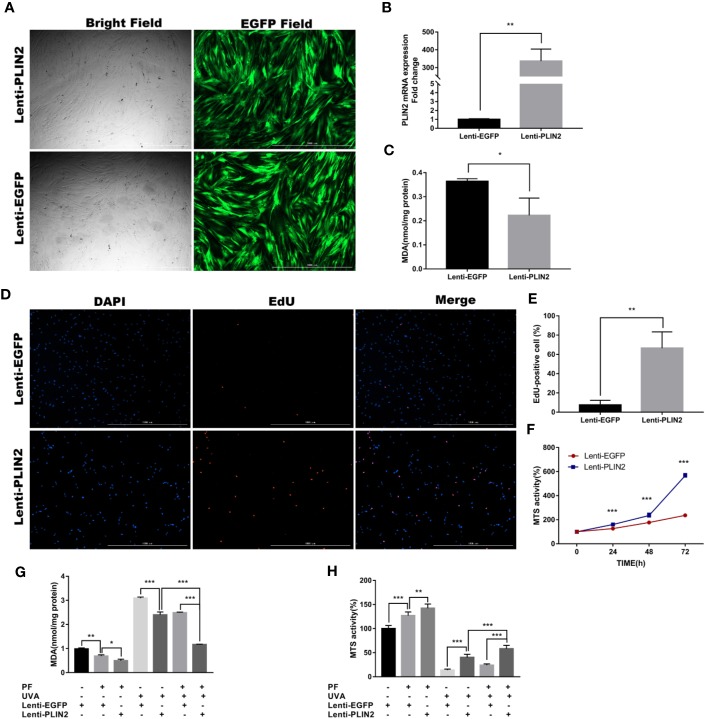
PLIN2 overexpression had a protective role in HDFs. **(A)** PLIN2 transfection was confirmed by observing EGFP positive cells under an inverted fluorescent microscope. **(B)** The transfection efficiency of PLIN2 was evaluated by RT-qPCR. **(C)** The level of MDA in PLIN2 overexpressed cells. **(D)** EdU staining was observed under fluorescence microscopy, red florescence indicated EdU-positive (EdU^+^) cells and blue florescence indicated cell nucleus, respectively. **(E)** The percentage of EdU^+^ cells in PLIN2 overexpressed cells. **(F)** The MTS activity of PLIN2 overexpressed cells and negative cells at the time points of 24, 48, and 72 h. **(G)** Detection of the level of MDA in cells treated with UVA+PLIN2 overexpression and/or UVA+PF-pretreatment. **(H)** The MTS activity of cells treated with UVA+PLIN2 overexpression and/or UVA+PF-pretreatment. All data were expressed as the mean ± SD according to at least three independent experiments. ^*^p < 0.05, ^**^p < 0.01, ^***^p < 0.001.

Having confirmed that PLIN2 may be upregulated by oxidative stress, we examined its function by overexpressing PLIN2 with lentiviral vector in fibroblasts. Successful transfection was confirmed by observing EGFP positive cells under an inverted fluorescent microscope (**Figure 5A**). The transfection efficiency was evaluated by RT-qPCR. Compared to the control cells, there was more than 300-fold change of increase in PLIN2 expression in transfected cells (p < 0.01) (**Figure 5B**). PLIN2 overexpressed cells have displayed significantly lower level of MDA compared with the negative control group (**Figure 5C**), suggesting PLIN2 may inhibit oxidative stress in HDFs, because MDA as a by-product of lipid peroxidation was known to be one of the manifestations of cell oxidative injury (Cini et al., 1994). EdU staining also revealed increased percentage of proliferating cells (EdU^+^) in PLIN2 overexpressed cells (**Figures 5D, E**). Additionally, MTS assay has demonstrated significant increase in MTS activity in PLIN2 overexpressed cells compared with the negative cells (**Figure 5F**). These results implied a protective role of PLIN2 in HDFs.

### Combination of PLIN2 Overexpression and PF Pre-Treatment Reduced UVA-Induced Injury in HDFs

Having demonstrated the promotive and inhibitory roles of PLIN2 in regulating the cell proliferation and MDA production, respectively, we presumed that PLIN2 may protect the fibroblasts from UVA induced cell damages. As shown in **Figures 5G, H**, PLIN2 overexpressed cells displayed significantly higher levels of MTS activity and lower level of MDA production compared with UVA radiation cells. PLIN2 overexpression combined with PF pre-treatment slightly reduced MDA level and increased MTS activity in HDFs. More importantly, this combination obviously enhanced the cytoprotective function of single agent alone by showing even less reductions in MTS activity and MDA generation induced by UVA radiation. These results might reveal the additive protective functions of PF treatment and PLIN2 overexpression against UVA induced injury, implying potential therapeutic effect of combining PLIN2 overexpression and PF in treating UVA induced photodamages in skin cells.

## Discussion

Photodamages caused by UVA radiation induced oxidative injuries are closely related to photoaging and skin cancer (D’Orazio et al., 2013; Gilchrest, 2013; Kammeyer and Luiten, 2015; Brem et al., 2017). In recent years, natural compounds have gained concerns in the development of skincare products and sunscreens to weaken photodamages. The present study has found that UVA induced cytotoxicity was inhibited by PF *via* Nrf2/HO-1/NQ-O1 signaling pathway or by PLIN2, and combining PLIN2 overexpression and PF demonstrated additive effect against UVA-related oxidative stress.

In recent years, natural antioxidants have attracted attentions in the development of cosmetic products against photodamages due to their antioxidative potency and low toxicity. For instance, resveratrol and carotenoid such as astaxanthin have been confirmed to protect skin against ultraviolet radiation (Sies and Stahl, 2004; Suganuma et al., 2010; Sharma et al., 2019). PF has been reported to attenuate UVB-induced cells apoptosis and DNA damage in human or hairless mouse skin keratinocytes (Lee et al., 2006; Kong et al., 2016). It appeared to be an antioxidant that protected human retinal pigment epithelium, rat pheochromocytoma cells, and human pulmonary endothelial cells from oxidative stress (Mao et al., 2010; Wankun et al., 2011; Yu et al., 2013). Our study has investigated the protective efficacy of PF against UVA radiation induced oxidative stress in HDFs. To our knowledge, this is the first time PF has been reported to be related to UVA radiation.

UVA radiation induced accumulated Nrf2 is essential in cell protection against oxidative stress (Hirota et al., 2005; Zhong et al., 2010; Tian et al., 2011; Hayes and Dinkova-Kostova, 2014; Yamamoto et al., 2018), and PF could inhibit oxidative injury by activating Nrf2 (Yu et al., 2013; Yang et al., 2016). Accordingly, our study has shown that in response to either UVA exposure or PF treatment, Nrf2 and its target genes HO-1 and NQ-O1 were significantly increased, and the expression levels of these three proteins were even higher in fibroblasts treated with PF+UVA compared to UVA alone. In absence of Nrf2, PF treatment lost the cytoprotective property against UVA radiation, and the capability of upregulating the expression of NQ-O1 and HO-1. These results have confirmed that PF regulated UVA induced oxidative stress *via* Nrf2.

As shown in our study, PLIN2 could be increased by oxidative stress induced by UVA or Nrf2 silencing, and PLIN2 overexpression could conversely inhibit oxidative stress and promote cell proliferation in fibroblasts. This might possibly suggest the potential antioxidant potency of PLIN2. Consistent with our results, PLIN2 has also been reported to be upregulated by ROS in HepG2 Cells (Jin et al., 2018). We speculated that PLIN2 may have exerted its antioxidative function *via* promoting the formation of LDs in HDFs, as PLIN2 has been previously reported to induce LD formation in macrophages and murine fibroblasts (Imamura et al., 2002; Chen et al., 2010), and PLIN2 induced LDs formation or lipid storage could reduce the levels of ROS under the condition of oxidative stress (Bensaad et al., 2014; Bailey et al., 2015; Cadenas et al., 2019). The underlying mechanisms of PLIN2 protecting fibroblasts and inhibiting oxidative stress following UVA radiation will be studied in the future.

**Figure 6:**
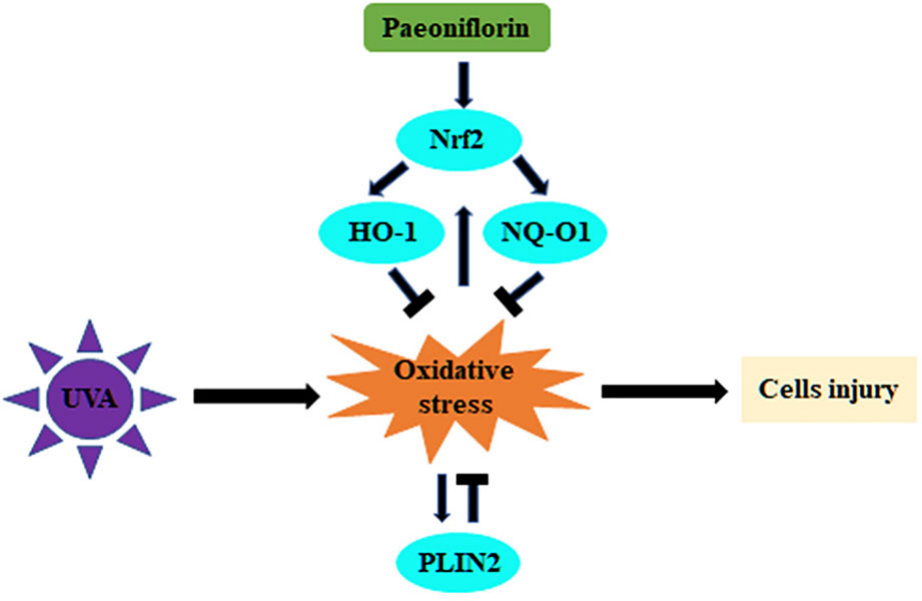
Schematic illustration of this study showing the regulation of UVA-related oxidative stress by PF *via* Nrf2/HO-1/NQ-O1 signaling pathway or by PLIN2.

Both PF and PLIN2 have demonstrated protective effects on fibroblasts, but interestingly, PF pre-treatment could reduce the PLIN2 expression after UVA radiation. This may be a result that PLIN2 compensated with Nrf2 in the regulation of oxidative stress. Therefore, when PF produced enough Nrf2 to effectively protect cells against UVA induced oxidative stress, less PLIN2 might be required by fibroblasts. Alternatively, PF was no longer able to inhibit oxidative stress when Nrf2 was knocked down and more PLIN2 might be produced by fibroblasts to exert cytoprotective function against oxidative stress (**Figure 6**). Given that both PF and PLIN2 have protective roles in fibroblasts, cells were treated by PLIN2 overexpression plus PF before UVA radiation. It was not surprised to see that the MDA was reduced and the MTS activity was improved, indicating that the combination of PLIN2 overexpression and PF pre-treatment exhibited additive anti-ultraviolet effects. The changes of MDA level and MTS activity were more obvious in UVA-radiated cells than UVA-spared cells, possibly because PLIN2 and PF played a greater protective role under the condition of greater oxidative stress. Our results provided a potential therapeutic strategy in treating UVA induced photodamages in skin cells.

In conclusion, our study demonstrated that PF inhibited UVA induced photodamage *via* Nrf2/HO-1/NQ-O1 signaling pathway, and combining PLIN2 overexpression and PF might have an additive protective effect against UVA radiation. The compensatory protections against UVA induced oxidative stress by PF and PLIN2 identified in this study have improved our understanding of the mechanisms of antioxidation, and may provide potential antiphotodamage therapeutic strategy. Further investigations on the underlying protective mechanisms of PLIN2 will be performed in the future work.

## Data Availability Statement

All datasets generated for this study are included in the article/supplementary material.

## Ethics Statement

The studies involving human participants were reviewed and approved by Ethics Committee of Medical Science Research, the First Hospital of China Medical University. Ethics number: AF-SOP-07-1.0-01. Written informed consent to participate in this study was not required in accordance with local/national guidelines.

## Author Contributions

Y-SL, YW, and H-XW contributed conception and design of the study. Y-SL, J-PY, S-BJ, P-YZ, YY, R-QQ, and Y-ZS analyzed the data. YJ performed the statistical analysis. Y-SL wrote the manuscript. YW and H-XW participated in revising the manuscript. TL provided the circumcised foreskins. YW, X-HG, and H-DC provided approval for publication of the content. All authors contributed to manuscript revision, read, and approved the submitted version.

## Funding

This work was supported by grants from the National Natural Science Fund (Grant number 81972940 and 81602741), Liaoning Province Natural Science Fund (Grant number 2019-ZD-0763).

## Conflict of Interest

The authors declare that the research was conducted in the absence of any commercial or financial relationships that could be construed as a potential conflict of interest.

## References

[B1] Bachelor M. A. Bowden G. T. (2004). ‘UVA-mediated activation of signaling pathways involved in skin tumor promotion and progression’. Semin. Cancer Biol. 14, 131–138. 10.1016/j.semcancer.2003.09.017 15018897

[B2] Bailey A. P. Koster G. Guillermier C. Hirst E. M. MacRae J. I. Lechene C. P. (2015). ‘Antioxidant Role for Lipid Droplets in a Stem Cell Niche of Drosophila’. Cell 163, 340–353. 10.1016/j.cell.2015.09.020 26451484 PMC4601084

[B3] Baird L. Dinkova-Kostova A. T. (2011). ‘The cytoprotective role of the Keap1-Nrf2 pathway’. Arch. Toxicol. 85, 241–272. 10.1007/s00204-011-0674-5 21365312

[B4] Bensaad K. Favaro E. Lewis C. A. Peck B. Lord S. Collins J. M. (2014). ‘Fatty acid uptake and lipid storage induced by HIF-1alpha contribute to cell growth and survival after hypoxia-reoxygenation’. Cell Rep. 9, 349–365. 10.1016/j.celrep.2014.08.056 25263561

[B5] Brem R. Guven M. Karran P. (2017). ‘Oxidatively-generated damage to DNA and proteins mediated by photosensitized UVA’. Free Radic. Biol. Med. 107, 101–109. 10.1016/j.freeradbiomed.2016.10.488 27989755 PMC5462485

[B6] Cadenas C. Vosbeck S. Edlund K. Grgas K. Madjar K. Hellwig B. (2019). ‘LIPG-promoted lipid storage mediates adaptation to oxidative stress in breast cancer’. Int. J. Cancer 145, 901–915. 10.1002/ijc.32138 30653260 PMC6618071

[B7] Chen F. Lu H. T. Jiang Y. (2004). ‘Purification of paeoniflorin from Paeonia lactiflora Pall. by high-speed counter-current chromatography’. J. Chromatogr. A 1040, 205–208. 10.1016/j.chroma.2004.04.023 15230527

[B8] Chen F. L. Yang Z. H. Wang X. C. Liu Y. Yang Y. H. Li L. X. (2010). ‘Adipophilin affects the expression of TNF-alpha, MCP-1, and IL-6 in THP-1 macrophages’. Mol. Cell Biochem. 337, 193–199. 10.1007/s11010-009-0299-7 19851831

[B9] Chen X. Wang K. Cederbaum A. I. Lu Y. (2019). ‘Suppressed hepatocyte proliferation via a ROS-HNE-P21 pathway is associated with nicotine- and cotinine-enhanced alcoholic fatty liver in mice’. Biochem. Biophys. Res. Commun. 512, 119–124. 10.1016/j.bbrc.2019.03.021 30876690 PMC6433518

[B10] Chignell C. F. Sik R. H. (2003). ‘A photochemical study of cells loaded with 2’,7’-dichlorofluorescin: implications for the detection of reactive oxygen species generated during UVA irradiation’. Free Radic. Biol. Med. 34, 1029–1034. 10.1016/S0891-5849(03)00022-4 12684087

[B11] Cini M. Fariello R. G. Bianchetti A. Moretti A. (1994). ‘Studies on lipid peroxidation in the rat brain’. Neurochem. Res. 19, 283–288. 10.1007/BF00971576 8177367

[B12] D’Orazio J. Jarrett S. Amaro-Ortiz A. Scott T. (2013). ‘UV radiation and the skin’. Int. J. Mol. Sci. 14, 12222–12248. 10.3390/ijms140612222 23749111 PMC3709783

[B13] Dolat E. Salarabadi S. S. Layegh P. Jaafari M. R. Sazgarnia S. Sazgarnia A. (2020). ‘The effect of UV radiation in the presence of TiO2-NPs on Leishmania major promastigotes’. Biochim. Biophys. Acta Gen. Subj. 1864, 129558. 10.1016/j.bbagen.2020.129558 32061714

[B14] Gilchrest B. A. (2013). ‘Photoaging’. J. Invest. Dermatol. 133, E2–E6. 10.1038/skinbio.2013.176 23820721

[B15] Graffmann N. Ring S. Kawala M. A. Wruck W. Ncube A. Trompeter H. I. (2016). ‘Modeling Nonalcoholic Fatty Liver Disease with Human Pluripotent Stem Cell-Derived Immature Hepatocyte-Like Cells Reveals Activation of PLIN2 and Confirms Regulatory Functions of Peroxisome Proliferator-Activated Receptor Alpha’. Stem Cells Dev. 25, 1119–1133. 10.1089/scd.2015.0383 27308945 PMC4971413

[B16] Hayes J. D. Dinkova-Kostova A. T. (2014). ‘The Nrf2 regulatory network provides an interface between redox and intermediary metabolism’. Trends Biochem. Sci. 39, 199–218. 10.1016/j.tibs.2014.02.002 24647116

[B17] Hirota A. Kawachi Y. Itoh K. Nakamura Y. Xu X. Banno T. (2005). ‘Ultraviolet A irradiation induces NF-E2-related factor 2 activation in dermal fibroblasts: protective role in UVA-induced apoptosis’. J. Invest. Dermatol. 124, 825–832. 10.1111/j.0022-202X.2005.23670.x 15816842

[B18] Hseu Y. C. Chou C. W. Senthil Kumar K. J. Fu K. T. Wang H. M. Hsu L. S. (2012). ‘Ellagic acid protects human keratinocyte (HaCaT) cells against UVA-induced oxidative stress and apoptosis through the upregulation of the HO-1 and Nrf-2 antioxidant genes’. Food Chem. Toxicol. 50, 1245–1255. 10.1016/j.fct.2012.02.020 22386815

[B19] Hseu Y. C. Lo H. W. Korivi M. Tsai Y. C. Tang M. J. Yang H. L. (2015). ‘Dermato-protective properties of ergothioneine through induction of Nrf2/ARE-mediated antioxidant genes in UVA-irradiated Human keratinocytes’. Free Radic. Biol. Med. 86, 102–117. 10.1016/j.freeradbiomed.2015.05.026 26021820

[B20] Hwang J. Y. Yadav A. K. Jang B. C. Kim Y. C. (2019). ‘Antioxidant and cytoprotective effects of Stachys riederi var. japonica ethanol extract on UVAirradiated human dermal fibroblasts’. Int. J. Mol. Med. 43, 1497–1504. 10.3892/ijmm.2019.4048 30628642

[B21] Imamura M. Inoguchi T. Ikuyama S. Taniguchi S. Kobayashi K. Nakashima N. (2002). ‘ADRP stimulates lipid accumulation and lipid droplet formation in murine fibroblasts’. Am. J. Physiol. Endocrinol. Metab. 283, E775–E783. 10.1152/ajpendo.00040.2002 12217895

[B22] Jin Y. Tan Y. Chen L. Liu Y. Ren Z. (2018). ‘Reactive Oxygen Species Induces Lipid Droplet Accumulation in HepG2 Cells by Increasing Perilipin 2 Expression’. Int. J. Mol. Sci. 19 (11), 3445. 10.3390/ijms19113445 PMC6274801 30400205

[B23] Kammeyer A. Luiten R. M. (2015). ‘Oxidation events and skin aging’. Ageing Res. Rev. 21, 16–29. 10.1016/j.arr.2015.01.001 25653189

[B24] Kim J. Cha Y. N. Surh Y. J. (2010). ‘A protective role of nuclear factor-erythroid 2-related factor-2 (Nrf2) in inflammatory disorders’. Mutat. Res. 690, 12–23. 10.1016/j.mrfmmm.2009.09.007 19799917

[B25] Kong L. Wang S. Wu X. Zuo F. Qin H. Wu J. (2016). ‘Paeoniflorin attenuates ultraviolet B-induced apoptosis in human keratinocytes by inhibiting the ROS-p38-p53 pathway’. Mol. Med. Rep. 13, 3553–3558. 10.3892/mmr.2016.4953 26936104

[B26] Kulms D. Zeise E. Poppelmann B. Schwarz T. (2002). ‘DNA damage, death receptor activation and reactive oxygen species contribute to ultraviolet radiation-induced apoptosis in an essential and independent way’. Oncogene 21, 5844–5851. 10.1038/sj.onc.1205743 12185583

[B27] Lee S. Lim J. M. Jin M. H. Park H. K. Lee E. J. Kang S. (2006). ‘Partially purified paeoniflorin exerts protective effects on UV-induced DNA damage and reduces facial wrinkles in human skin’. J. Cosmet. Sci. 57, 57–64. 16676123

[B28] Liu Y. Han J. Zhou Z. Li D. (2019). ‘Paeoniflorin protects pancreatic beta cells from STZ-induced damage through inhibition of the p38 MAPK and JNK signaling pathways’. Eur. J. Pharmacol. 853, 18–24. 10.1016/j.ejphar.2019.03.025 30880178

[B29] Magne J. Aminoff A. Perman Sundelin J. Mannila M. N. Gustafsson P. Hultenby K. (2013). ‘The minor allele of the missense polymorphism Ser251Pro in perilipin 2 (PLIN2) disrupts an alpha-helix, affects lipolysis, and is associated with reduced plasma triglyceride concentration in humans’. FASEB J. 27, 3090–3099. 10.1096/fj.13-228759 23603836

[B30] Mao Q. Q. Zhong X. M. Feng C. R. Pan A. J. Li Z. Y. Huang Z. (2010). ‘Protective effects of paeoniflorin against glutamate-induced neurotoxicity in PC12 cells via antioxidant mechanisms and Ca(2+) antagonism’. Cell Mol. Neurobiol. 30, 1059–1066. 10.1007/s10571-010-9537-5 20577899 PMC11498830

[B31] Marrot L. Jones C. Perez P. Meunier J. R. (2008). ‘The significance of Nrf2 pathway in (photo)-oxidative stress response in melanocytes and keratinocytes of the human epidermis’. Pigment Cell Melanoma Res. 21, 79–88. 10.1111/j.1755-148X.2007.00424.x 18353146

[B32] Meewes C. Brenneisen P. Wenk J. Kuhr L. Ma W. Alikoski J. (2001). ‘Adaptive antioxidant response protects dermal fibroblasts from UVA-induced phototoxicity’. Free Radic. Biol. Med. 30, 238–247. 10.1016/S0891-5849(00)00463-9 11165870

[B33] Pinnell S. R. (2003). ‘Cutaneous photodamage, oxidative stress, and topical antioxidant protection’. J. Am. Acad. Dermatol. 48, 1–19; quiz 20-2. 10.1067/mjd.2003.16 12522365

[B34] Raval C. M. Zhong J. L. Mitchell S. A. Tyrrell R. M. (2012). ‘The role of Bach1 in ultraviolet A-mediated human heme oxygenase 1 regulation in human skin fibroblasts’. Free Radic. Biol. Med. 52, 227–236. 10.1016/j.freeradbiomed.2011.10.494 22107958

[B35] Roberts J. E. Wielgus A. R. Boyes W. K. Andley U. Chignell C. F. (2008). ‘Phototoxicity and cytotoxicity of fullerol in human lens epithelial cells’. Toxicol. Appl. Pharmacol. 228, 49–58. 10.1016/j.taap.2007.12.010 18234258 PMC2358981

[B36] Saewan N. Jimtaisong A. (2015). ‘Natural products as photoprotection’. J. Cosmet. Dermatol. 14, 47–63. 10.1111/jocd.12123 25582033

[B37] Sasaki H. Akamatsu H. Horio T. (2000). ‘Protective role of copper, zinc superoxide dismutase against UVB-induced injury of the human keratinocyte cell line HaCaT’. J. Invest. Dermatol. 114, 502–507. 10.1046/j.1523-1747.2000.00914.x 10692109

[B38] Schafer M. Werner S. (2015). ‘Nrf2–A regulator of keratinocyte redox signaling’. Free Radic. Biol. Med. 88, 243–252. 10.1016/j.freeradbiomed.2015.04.018 25912479

[B39] Sharma B. Iqbal B. Kumar S. Ali J. Baboota S. (2019). ‘Resveratrol-loaded nanoemulsion gel system to ameliorate UV-induced oxidative skin damage: from in vitro to in vivo investigation of antioxidant activity enhancement’. Arch. Dermatol. Res. 311, 773–793. 10.1007/s00403-019-01964-3 31432208

[B40] Shou Q. Jin L. Lang J. Shan Q. Ni Z. Cheng C. (2018). ‘Integration of Metabolomics and Transcriptomics Reveals the Therapeutic Mechanism Underlying Paeoniflorin for the Treatment of Allergic Asthma’. Front. Pharmacol. 9, 1531. 10.3389/fphar.2018.01531 30761008 PMC6362974

[B41] Sies H. Stahl W. (2004). ‘Carotenoids and UV protection’. Photochem. Photobiol. Sci. 3, 749–752. 10.1039/b316082c 15295630

[B42] Son S. H. Goo Y. H. Choi M. Saha P. K. Oka K. Chan L. C. (2016). ‘Enhanced atheroprotection and lesion remodelling by targeting the foam cell and increasing plasma cholesterol acceptors’. Cardiovasc. Res. 109, 294–304. 10.1093/cvr/cvv241 26487692 PMC4724936

[B43] Suganuma K. Nakajima H. Ohtsuki M. Imokawa G. (2010). ‘Astaxanthin attenuates the UVA-induced up-regulation of matrix-metalloproteinase-1 and skin fibroblast elastase in human dermal fibroblasts’. J. Dermatol. Sci. 58, 136–142. 10.1016/j.jdermsci.2010.02.009 20219323

[B44] Surh Y. J. (2003). ‘Cancer chemoprevention with dietary phytochemicals’. Nat. Rev. Cancer 3, 768–780. 10.1038/nrc1189 14570043

[B45] Tian F. F. Zhang F. F. Lai X. D. Wang L. J. Yang L. Wang X. (2011). ‘Nrf2-mediated protection against UVA radiation in human skin keratinocytes’. Biosci. Trends 5, 23–29. 10.5582/bst.2011.v5.1.23 21422597

[B46] Wang J. S. Huang Y. Zhang S. Yin H. J. Zhang L. Zhang Y. H. (2019). ‘A Protective Role of Paeoniflorin in Fluctuant Hyperglycemia-Induced Vascular Endothelial Injuries through Antioxidative and Anti-Inflammatory Effects and Reduction of PKCbeta1’. Oxid. Med. Cell Longev. 2019, 5647219. 10.1155/2019/5647219 31093316 PMC6481012

[B47] Wankun X. Wenzhen Y. Min Z. Weiyan Z. Huan C. Wei D. (2011). ‘Protective effect of paeoniflorin against oxidative stress in human retinal pigment epithelium in vitro’. Mol. Vis. 17, 3512–3522. 22219646 PMC3249435

[B48] Yamamoto M. Kensler T. W. Motohashi H. (2018). ‘The KEAP1-NRF2 System: a Thiol-Based Sensor-Effector Apparatus for Maintaining Redox Homeostasis’. Physiol. Rev. 98, 1169–1203. 10.1152/physrev.00023.2017 29717933 PMC9762786

[B49] Yang X. Yao W. Shi H. Liu H. Li Y. Gao Y. (2016). ‘Paeoniflorin protects Schwann cells against high glucose induced oxidative injury by activating Nrf2/ARE pathway and inhibiting apoptosis’. J. Ethnopharmacol. 185, 361–369. 10.1016/j.jep.2016.03.031 26979341

[B50] Yang H. L. Lee C. L. Korivi M. Liao J. W. Rajendran P. Wu J. J. (2018). ‘Zerumbone protects human skin keratinocytes against UVA-irradiated damages through Nrf2 induction’. Biochem. Pharmacol. 148, 130–146. 10.1016/j.bcp.2017.12.014 29273513

[B51] Yin J. J. Liu J. Ehrenshaft M. Roberts J. E. Fu P. P. Mason R. P. (2012). ‘Phototoxicity of nano titanium dioxides in HaCaT keratinocytes–generation of reactive oxygen species and cell damage’. Toxicol. Appl. Pharmacol. 263, 81–88. 10.1016/j.taap.2012.06.001 22705594 PMC3407290

[B52] Yu J. Zhu X. Qi X. Che J. Cao B. (2013). ‘Paeoniflorin protects human EA.hy926 endothelial cells against gamma-radiation induced oxidative injury by activating the NF-E2-related factor 2/heme oxygenase-1 pathway’. Toxicol. Lett. 218, 224–234. 10.1016/j.toxlet.2013.01.028 23403272

[B53] Yu C. Fan X. Li Z. Liu X. Wang G. (2017). ‘Efficacy and safety of total glucosides of paeony combined with acitretin in the treatment of moderate-to-severe plaque psoriasis: a double-blind, randomised, placebo-controlled trial’. Eur. J. Dermatol. 27, 150–154. 10.1684/ejd.2016.2946 28400341

[B54] Zhang W. Dai S. M. (2012). ‘Mechanisms involved in the therapeutic effects of Paeonia lactiflora Pallas in rheumatoid arthritis’. Int. Immunopharmacol. 14, 27–31. 10.1016/j.intimp.2012.06.001 22705050

[B55] Zhao B. He Y. Y. Chignell C. F. Yin J. J. Andley U. Roberts J. E. (2009). ‘Difference in phototoxicity of cyclodextrin complexed fullerene [(gamma-CyD)2/C60] and its aggregated derivatives toward human lens epithelial cells’. Chem. Res. Toxicol. 22, 660–667. 10.1021/tx800478u 19281132 PMC2800100

[B56] Zhong J. L. Edwards G. P. Raval C. Li H. Tyrrell R. M. (2010). ‘The role of Nrf2 in ultraviolet A mediated heme oxygenase 1 induction in human skin fibroblasts’. Photochem. Photobiol. Sci. 9, 18–24. 10.1039/B9PP00068B 20062840

